# Missed diagnostic opportunities and English general practice: a study to determine their incidence, confounding and contributing factors and potential impact on patients through retrospective review of electronic medical records

**DOI:** 10.1186/s13012-015-0296-z

**Published:** 2015-07-29

**Authors:** Sudeh Cheraghi-Sohi, Hardeep Singh, David Reeves, Jill Stocks, Morris Rebecca, Aneez Esmail, Stephen Campbell, Carl de Wet

**Affiliations:** NIHR Greater Manchester Primary Care Patient Safety Translational Research Centre, University of Manchester, 7th Floor: Williamson Building, Manchester, M13 9PL UK; Centre for Primary Care: Institute of Population Health, University of Manchester, 7th Floor: Williamson Building, Manchester, M13 9PL UK; Houston Veterans Affairs Centre for Innovations in Quality, Effectiveness and Safety, Michael E. DeBakey Veterans Affairs Medical Centre and Baylor College of Medicine, 2002 Holcombe Blvd. 152, Houston, TX 77030, 713.794.8601 USA; Centre for Research and Action in Public Health (CeRAPH), University of Canberra, Building 22, Floor B, University Drive, Bruce, ACT 2617 Australia; School of Medicine, Gold Coast Campus, Griffith University, Queensland, Australia

**Keywords:** Patient safety, Primary care, Diagnoses, Missed diagnostic opportunities, Diagnostic error, General practice

## Abstract

**Background:**

Patient safety research has focused largely on hospital settings despite the fact that in many countries, the majority of patient contacts are in primary care. The knowledge base about patient safety in primary care is developing but sparse and diagnostic error is a relatively understudied and an unmeasured area of patient safety. Diagnostic error rates vary according to how ‘error’ is defined but one suggested hallmark is clear evidence of ‘missed opportunity’ (MDOs) makes a correct or timely diagnosis to prevent them. While there is no agreed definition or method of measuring MDOs, retrospective manual chart or patient record reviews are a ‘gold standard’. This study protocol aims to (1) determine the incidence of MDOs in English general practice, (2) identify the confounding and contributing factors that lead to MDOs and (3) determine the (potential) impact of the detected MDOs on patients.

**Methods/Design:**

We plan to conduct a two-phase retrospective review of electronic health records in the Greater Manchester (GM) area of the UK. In the first phase, clinician reviewers will calibrate their performance in identifying and assessing MDOs against a gold standard ‘primary reviewer’ through the use of ‘double’ reviews of records. The findings will enable a preliminary estimate of the incidence of MDOs in general practice, which will be used to calculate the number of records to be reviewed in the second phase in order to estimate the true incidence of MDO in general practice. A sample of 15 general practices is required for phase 1 and up to 35 practices for phase 2. In each practice, the sample will consist of 100 patients aged ≥18 years on 1 April 2013 who have attended a face-to-face ‘index consultation’ between 1 April 2013 and 31 March 2015. The index consultation will be selected randomly from each unique patient record, occurring between 1 July 2013 and 30 June 2014.

**Discussion:**

There are no reliable estimates of safety problems related to diagnosis in English general practice. This study will lay the foundation for safety improvements in this area by providing a more reliable estimate of MDOs, their impact and their contributory factors.

**Electronic supplementary material:**

The online version of this article (doi:10.1186/s13012-015-0296-z) contains supplementary material, which is available to authorized users.

## Background

Patient safety has received considerable global attention since the publication of the landmark report ‘To Err is Human’ in 1999 [1]. This attention has however been focused largely toward hospital settings leading to the identification of a so called ‘lost decade’ with respect to settings such as primary care [2]. This is despite the fact that in many countries, including the United Kingdom (UK), the majority of patient contacts are with health care providers in primary care. The knowledge base about patient safety in this setting is still sparse [3] and some may still perceive primary care as a low-risk environment. However, a recent review estimated that there may be a patient safety incident in approximately 2 % of all family practice consultations [4]. Moreover, the sheer volume of patient contacts (approximately 340 million consultations per year in the UK [5]) multiplies opportunities for errors. A recent systematic review of tools that can be used by family practitioners as part of a patient safety toolkit found that tools for diagnostic error accounts for <1 % of total literature [6]. Diagnostic error remains relatively understudied and unmeasured area of patient safety [7].

### Diagnostic error

Data about the incidence, types and causes of errors encountered in primary care are scant and there are no large-scale epidemiological studies in the UK to reliably quantify error and harm rates in this setting. However, among the different types of errors, medication-related errors and diagnostic errors (DEs) are the most common in primary care [8, 9]. Despite their potential and actual significant impact, DE have received less attention relative to other types of error even though there is evidence to suggest that DEs are more common in primary than in secondary care settings [9–11]. DE-associated harm has been estimated at 0.1 % of primary care diagnoses, but the overall DE rate may be as high as 15 % [12]. Recent work in the United States (US) combining data from three large observational studies estimated that 5 % of US adults will experience a DE every year in the outpatient setting, but comparative data from the UK is lacking. DE rates vary according to different diseases and their myriad clinical presentations, with atypical and non-specific presentations substantially increasing the risk of DE. However, no single disease accounts for more than 5 % of malpractice claims [13].

### Defining diagnostic error

DE rates are likely to vary substantially according to the criteria used to define ‘error’. A central issue in the reliable measurement of DE is therefore how it is defined. Some studies define diagnostic error as any diagnosis that is (i) wrong, e.g. another diagnosis was made before the correct one; (ii) delayed, e.g. insufficient information was available to make an earlier diagnosis; or (iii) missed, e.g. no diagnosis was ever made. The decision as to whether a diagnostic error occurred or not is judged from a subsequent assessment of more definitive information, regardless of whether an error in the diagnostic process occurred [14, 15]. To address this potential limitation, it has recently been argued that the definition of a diagnostic error should include the requisite of clear evidence of ‘missed opportunities’ to prevent them [16]. There are three specific aspects to a ‘missed opportunity’:

1. Case analysis reveals evidence of a missed opportunity to make a correct or timely diagnosis. The concept of a missed opportunity implies that something different could have been done to make the correct diagnosis earlier. The missed opportunity may result from cognitive and/or system factors or may be attributable to more obvious factors, such as lapses in accountability or clear evidence of liability or negligence.
2. A missed opportunity is framed within the context of an ‘evolving’ diagnostic process. The determination of error depends on the temporal or sequential context of events. Evidence of omission (failure to do the right thing) or commission (doing something wrong) exists at the particular point in time at which the ‘error’ occurred.
3. The opportunity could be missed by the provider, care team, system, and/or patient. A preventable error or delay in diagnosis may occur due to factors outside the clinician’s immediate control or when a clinician’s performance is not contributory [16].

In this study, we define and conceptualise diagnostic errors therefore as ‘missed diagnostic opportunities’ (MDOs).

### Aetiology and impact of MDOs

Research suggests that the aetiology of the vast majority of MDOs is multi-factorial and arises via a complex interplay of system and cognitive contributory factors [14]. One potentially useful classification identifies five interactive process dimensions: (i) the patient-provider encounter (history, exam, ordering tests/referrals based on assessment); (ii) performance and interpretation of diagnostic tests; (iii) follow-up and tracking of diagnostic information over time; (iv) subspecialty and referral-specific issues; and (v) patient-related factors [17]. Regardless of their origins, diagnostic errors can have important consequences [18, 19]:

- Patients may suffer preventable harm, for example through conditions which remain untreated or by receiving inappropriate and unnecessary treatment;
- Doctors may be subject to malpractice claims (diagnostic error accounts for approximately two-thirds of malpractice claims against general practitioners (GPs) in the UK); and
- Additional financial costs are incurred by health care systems.

### Rationale for studying MDOs

The lack of progress in understanding and determining MDOs and in particular MDOs in primary care may be attributed to several factors. The diagnostic process often spans multiple health care settings and involves different professional staff groups, which in turn introduces myriad challenges, such as co-ordinating care and managing the timely and secure communication of patient information. Furthermore, diagnoses are made during time-pressured consultations within primary care settings, where providers are often remain unaware of ultimate patient outcomes [17]. As a result, it is hard to detect MDOs, ascertain their underlying causes and, ultimately, very difficult to prevent them [17]. Consequently, measuring MDOs and calculating a reliable ‘error rate’ is challenging. To date, the incidence of MDOs has been estimated using at least eight different methods [11]. Each method has its associated strengths and weaknesses, but retrospective manual chart or patient record reviews are considered the ‘gold standard’ [20]. Although laborious, the method allows for the overarching diagnostic process to be traced and account diagnoses that evolves over time.

Identifying, measuring and understanding MDOs in primary care is the first step in developing policies and interventions to reduce harm and improve patient safety in this area. At present, we have no reliable estimates of diagnostic error in English general practice with which to formulate any such policies or interventions, hence the need for this work.

### Aims and objectives

The study has three specific aims:

- To determine the incidence of MDOs in English general practice;
- To identify the confounding and contributing factors that lead to MDOs; and
- To determine the impact or potential impact of the detected MDOs on patients

The secondary objectives of the study are:

- To test for significant associations between MDO rates and specific patient, index consultation, practice and diagnostician variables.
- To compare the MDO rates of the participating practices and different professional groups.
- To describe the types of diagnoses clinicians made during the study period as well as the types of MDOs.

## Research team

### Methods

#### Study design

The study design is a retrospective review of electronic medical records that will be undertaken in two consecutive phases. In the first phase, clinician reviewers will gain experience and confidence in the review method and at the same time calibrate their performance in identifying and assessing MDOs against a gold standard ‘primary reviewer’ through the use of ‘double’ reviews of records. Thus, a primary goal of this phase is to maximise consistency of ratings across reviewers. The review findings from phase 1 will then be used to calculate a preliminary estimate of the incidence of MDOs in general practice. This preliminary rate will be used to calculate the number of records to be reviewed in the second phase in order to estimate the true incidence of MDO in general practice to a specified degree of precision and reliability. In other words, the MDO rate from phase 1 will ensure that no more than the required number of records is reviewed during phase 2. This is an essential requirement of the study to ensure that the available resources are used efficiently.

#### Setting

The study will be conducted in the Greater Manchester (GM) area. More specifically, the electronic patient records of general practices in the GM Clinical Commissioning Groups (CCGs) will be considered for potential inclusion. However, practice staff and patients will not directly be involved in any aspect of the study.

#### Samples

i. The sampling strategy will involve random selection of sets of electronic health records from a stratified sample of general practices.

#### General practices

A sample of 15 general practices is required for phase 1 of the review process. An additional sample of 35 practices may be required for phase 2. However, this is an estimate that will be revised as described in ‘Calculation of sample size’ section below.

Purposeful sampling will be used to select a sample of practices stratified by size and neighbourhood deprivation. Practice size will be based on the number of registered patients and coded as ‘large’, ‘medium’ or ‘small’. Neighbourhood deprivation will be based on the national quintiles of the English Index of Multiple Deprivation (IMD) for 2010 [21], based on the postcode of the general practice. In addition, a pragmatic requirement for suitable practices will be availability of space for the administrator and clinician reviewers.

ii. Electronic health records

Electronic health records for 100 unique patients that meet the following four inclusion criteria will be randomly sampled from each participating practice: (1) age ≥18 years old on the 1 April 2013; (2) registered continuously with the same practice from 1 April 2013 to 31 March 2015 (thus excluding records of temporary residents); (3) availability of the complete medical record for review; and (4) must have attended a face-to-face ‘index consultation’ during this period.

Each patient’s index consultation represents the ‘anchor’ of the review process and marks the reviewers’ starting point for that patient. A single index consultation will be randomly selected from each unique patient record, occurring within the 12-month period commencing on 1 July 2013 and ending on 30 June 2014. Across the patient sample within each practice, the index consultation dates will be spread over the course of the defined 12-month period to account for potential seasonal effects on reasons for consulting and hence MDOs. This will be done by assigning four week-long sampling ‘windows’ to the practice and randomly selecting 25 index consultations from all patient consultations within each window. The first sampling window will be randomly chosen from the first 13 weeks of the period and each subsequent window will be 13 weeks later.

#### Reviewers

Five GPs will be recruited and trained as clinician reviewers and will meet the following inclusion criteria: (1) currently working as a GP or has worked as a GP in the last 12 months; and (2) a minimum of 5 years’ experience as a GP. Based on previous experience and knowledge of safe practice, one of the five GPs will be designated the ‘primary reviewer’ and will undergo additional training and calibration with clinical members of the project research team group. The primary reviewer will then act as the gold standard against which the performance of the four remaining GPs in identifying and evaluating MDOs will be calibrated.

#### Calculation of sample size

##### Phase 1 sample

The primary objectives of phase 1 are to calibrate the reviewers with the primary reviewer and to collect sufficient data to reliably estimate the sample required for phase 2. For these purposes, we have determined that a phase 1 sample of 15 practices and 100 records at each will be required. For calibration, the study uses overall sensitivity and specificity of the reviewers of 75 % or better as acceptable and 60 % or less as fully unacceptable, assessed using the latter 50 % of records (see ‘Data analysis’ section below). For an MDO rate of 5 % or higher and true sensitivity and specificity of 75 % or better, this sample will produce observed sensitivity and specificity with more than 99 % likelihood of being greater than 60 %: that is, with a less than 1 % chance that truly acceptable calibration will be mistaken as unacceptable, and vice versa. This calculation (and those below) also assumes that 20 % of records will contain no new diagnosis and therefore contribute no usable data (based on clinical experience).

The sample will also yield sufficiently precise estimates of both the overall MDO rate and of the degree of clustering of MDOs within practices (measured by the intra-cluster correlation coefficient (ICC)) to estimate the required sample at phase 2. Assuming a fairly high ICC of 0.05 (which for an overall MDO rate of 5 % implies that 95 % of individual practices have rates between 0 % and 15 %), this sample will yield an MDO rate with a 95 % confidence interval of 2.3–7.7 % when the true rate is 5 %; and 6.2–13.8 % when the true rate is 10 %.

##### Phase 2 sample

The number of electronic patient records to be sampled and reviewed at phase 2 will depend upon the estimates of the MDO rate and the ICC obtained from phase 1. In addition, to allow estimation of the relative rates of different forms of MDO, we aim to pick up a minimum of 100 MDOs in total across phase 1 and phase 2 combined (to give an error of +/−10 % on an MDO accounting for 50 % of all MDOs; +/−6 % on an MDO accounting for 10 %). As a pre-check that sample sizes will be sufficient for this purpose, we have made estimates for three true MDO rate scenarios: 1, 5 and 10 % (at an ICC of 0.05). The results are shown in Table 1.

**Table 1:** Sample sizes of records and practices as determined by different MDO rates

Scenario	True MDO rate (%)	Number of practices (phases 1 + 2)	Number of records per practice	Total number of records reviewed	Expected number of detected MDOs	95 % CI on the estimated MDO rate
1	1	50	250	12500	100	0.4-1.6
2	5	50	100	5000	200	3.5-6.5
3	10	40	100	4000	320	7.7-12.3

If the overall MDO rate from phase 1 is 5 % (Table 1, scenario 2), we will need to review a total of 5000 records, comprised of samples of 100 records selected from each of 50 general practices (15 at phase 1 plus 35 at phase 2). We calculate that this sample will yield approximately 200 MDOs and produce an estimate of the true MDO rate with error limits of at most 1.5 % (i.e. a 95 % confidence interval of 3.5–6.5 %). If the MDO rate at phase 1 is close to 1 %, we would instead need to review 250 records at each of 50 practices (12,500 in total) to obtain 100 MDOs (Table 1, scenario 1). To achieve this may be outside of the resources of the study hence if this is the case, we will revisit the aims of the main study.

#### Recruitment

Recruitment will commence as soon as we have obtained NHS Research Ethics Committee, Confidentiality Advisory Group and Research & Development (R&D) approvals for the study and will continue until we have recruited the requisite number of general practices as described in the ‘Samples’ section. We aim to recruit CCGs, general practices, an administrator and clinician reviewers.

##### CCGs

The recruitment process will begin by contacting the GM CCGs and meeting with their leaders in order to seek their support and approval for participation in this study. Each GM CCGs has many general practices with diverse geographical spread and wide-ranging socio-economic conditions and all of them will be considered once we have obtained senior leadership support.

##### General practices

We will send practices an introductory letter (Additional file 1) and further information about the study in an information sheet (Additional file 2) by post as well as a slip to allow practices to express an interest in participating, with pre-paid envelopes to return the consent forms (Additional file 3). The information sheet will outline the aims of the study, the method, potential risks and benefits of participating as well as the service support costs we can offer to practices. The recruitment letter will be signed by the Greater Manchester Primary Care Patient Safety Translational Research Centre (PSTRC) Principal Investigator (PI) and Medical Director of the relevant CCG. Two further reminders will be sent to those practices that do not respond at fortnightly intervals. Participating practices will be reimbursed £1000 for taking part.

#### Administrator

The administrator will be recruited from the Greater Manchester PSTRC and receive further training relevant to the study tasks. The main task of the administrator will be to compile a list of index consultations (one per record) in each participating practice as the first step in the review process.

#### Clinician reviewers

One reviewer will be recruited from each of the three participating CCGs if feasible. Two additional reviewers will be recruited, one as the ‘primary reviewer’ and the other as a ‘floater’ to allow for holidays and unforeseen eventualities in the lives of the other reviewers. The reviewers will be remunerated for their time in accordance with locum rates.

### Data collection

#### Data preparation

The administrator will visit each practice to create a list of ‘index consultations’ (one per patient record) and to complete the ‘patient demographic’ section on the data extraction form (DEF: Additional file 4) and separate ‘practitioner’ and ‘practice demographics’ sections on the DEF that was specifically designed for this study (Additional file 5). All relevant consultations for the 12-month period (1 July 2013 to 30 June 2014) will initially be identified by the administrator. From these, the required number (*n* = 100) of unique and eligible index consultations will be selected by using the Random Integer Set Generator.^1^

#### Period of review in each record

**Fig. 1:**
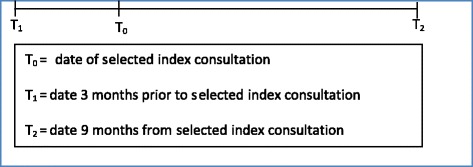
The minimum period of review in each record (12 months)

A minimum of 12 months will be reviewed in each record. More specifically, a period of 3 months before and 9 months after the selected index consultation in each record will be reviewed (Fig. 1). This timeframe is informed by the research team’s previous experience and is also similar to comparable studies of MDOs [22]. However, we acknowledge that some MDOs may only become apparent after a longer duration of time. Reviewers may therefore, at their own discretion, choose to extend the review period.

#### The four-step review process

The vast majority of data will be collected by the administrator and clinician reviewers using a structured, four-step review process and a DEF (Additional file 4) specifically designed for this study. The four consecutive steps are to:

1. Gather information about the index consultation;
2. Record the diagnosis or diagnoses that were made during the index consultation;
3. Gather information relating to the diagnosis or diagnoses;
4. Rate and describe the characteristics of the diagnosis or diagnoses and any MDOs

Further details about the four steps are available as an Additional file (Additional file 6) and the practical application of the steps are described in a coding manual (Additional file 6). Some of the main points are discussed below.

#### Step 1: Gather information about the index consultation

This part of the data collection will be performed by the administrator while steps 2–4 will be performed by the clinician reviewers. The following information will be extracted by the administrator from the index consultation in each record:

- The date, place (either home visit or at the practice) and time of day of the selected index consultation (08:00–11:59, 12:00–13:59; 14:00–18.30; or out-of-hours);
- The age of the patient on the day of the index consultation;
- The patient’s gender;

The following information will be extracted by the administrator from each record on the day the search is conducted:

- The number of repeat medication items prescribed;
- The type and number of documented morbidities

#### Step 2: Recording the diagnosis or diagnoses that were made during the index consultation

During this part of the data collection, the clinician reviewers collect information on the diagnosis or diagnoses that were made during the index consultation. If there were no diagnosis made, the review for that specific record is effectively over, and the reviewer continues with the next record.

#### Step 3: Gather information relating to the diagnosis or diagnoses

Reviewers will systematically gather information about the index consultation diagnosis or diagnoses in order to make professional judgments in step 4 about (i) the accuracy of each diagnosis and (ii) whether there had been missed diagnostic opportunities. The systematic approach involves searching for the presence of seven predefined ‘triggers’ or prompts in each record and considering eight questions about potential MDOs (Additional file 6), based on the work in the US by co-author Singh [16].

#### Step 4: Rate and describe the characteristics of the diagnosis or diagnoses and any MDOs

Reviewers will use their clinical experience and the information they gathered in step 3 to rate and record the following on the DEF for each record:

- The accuracy of each diagnosis;
- Whether there were MDOs, and their confidence in this finding;
- The potential impact of MDOs on patients;
- Factors that may have contributed to the MDOs;
- A description of the MDO;
- Any other relevant comments, reflections or feedback

#### Feasibility

Based on the steering group’s experience, we estimate that clinicians can review approximately 25 electronic medical records and complete a DEF for each in a 4-hour session.

#### Inter-rater agreement

We aim to achieve a high-level of inter-rater agreement between clinician reviewers by providing formal training, ongoing ‘expert’ and peer support and double coding and calibration during phase 1 of the review process, plus some monitoring to ensure continued calibration during phase 2.

#### Initial training and ongoing support

GP reviewers will receive 2 days of training, consisting of face-to-face presentations, opportunity to practice new skills with case studies, feedback on performance and participation in an open-forum discussion. In addition, they will be provided with a range of educational materials, including a coding manual (Additional file 6). The steering group will be available throughout the study period to provide support as required.

#### Phase 1 calibration and double coding

During phase 1, the primary reviewer will review every record in the sample. Each of the three other reviewers (reviewers 2–4) will be paired with the primary reviewer in 5 of the 15 practices (Table 2). A fifth reviewer has also been trained in case one or the main reviewers is unable to continue. The fifth reviewer will be calibrated to the gold standard across ten practices (rather than five) to ensure they could stand in for the primary reviewer themselves if the need arises. The order of the reviewers will be varied from practice to practice so that the initial reviewer is not always the same. The reviewers will complete the DEF for each record independently of each other before comparing their findings and ratings. Any differences will be resolved through discussion to reach consensus.

**Table 2:** Reviewer pairs in phase 1 of the review process.

Reviewer	Practice														
	1	2	3	4	5	6	7	8	9	10	11	12	13	14	15
1^a^	X	X	X	X	X	X	X	X	X	X	X	X	X	X	X
2	X			X			X			X			X		
3		X			X			X			X			X	
4			X			X			X			X			X
5	X	X	X	X	X	X	X	X	X	X					

^a^Primary reviewer

#### Phase 2 reviews

Assuming that an adequate level of calibration is achieved in phase 1, the records for each practice participating in phase 2 will be reviewed by one of the four reviewers only. However, the primary reviewer will double-check ten records from each of the other reviewers at every third practice they rate (making around an extra 150 reviews in all).

### Data analysis

For analysis purposes, the data from phases 1 and 2 will be pooled together, with the data for each phase 1 practice being the agreed final ratings between the two reviewers.

#### Descriptive analysis

Simple descriptive statistics, including means, standard deviations, medians and inter-quartile ranges, will be used to characterise the overall MDO rate and individual practice and subgroup rates, e.g. professional group and month of year. The precision of the MDO rate estimates from each analysis will be expressed in terms of the confidence interval around the rate. The overall rate of MDOs will be calculated by aggregating all practice data from phases 1 and 2. In addition, we will compare the distribution of the sample of practices in terms of key practice variables (e.g. list size, IMD) to all practices in GM and in England and derive weights from this in order to compute weighted estimates of the mean rates for these larger geographical areas.

Analysis of free-text data will be undertaken by the reviewers, research team and steering group. We will produce an output similar to Singh [23] to outline the symptoms and primary types of missed diagnostic opportunities. Symptoms and diagnoses will be coded and summarised using descriptive statistics.

#### Predictive factors

We will use multilevel logistic regression (records within clinicians within practices) to investigate a wide range of variables potentially associated with MDOs (Table 3). The analysis will be conducted according to an a priori analysis plan that fully specifies the outcome and explanatory factors, other covariates, treatment of missing values and methods of analysis.

**Table 3:** Variables potentially associated with MDOs

Main variable	Specific aspects to consider for analysis
Patient	Age
	Gender
	Number of chronic conditions
	Number of repeat medication items
Index consultation	Day of week
	Time of day
	Location of consultation (at home or in practice)
Diagnostician	Professional role (as recorded on Additional file 6)
	Gender (as recorded on Additional file 6)
	Age (as recorded on Additional file 6)
	Country in which professional qualifications were obtained (as recorded on the General Medical Council [GMC] register)
	Number of years of clinical experience (as recorded on the GMC register)
General practices	Size of practice, based on the number of registered patients (recorded on Additional file 6)
	Type of contract type (Personal Medical Services [PMS] v General Medical Services [GMS]) (recorded on Additional file 5: clinician and demographic form)
	Area of deprivation (as recorded by the English Indices of Multiple Deprivation (IMD) https://www.gov.uk/government/statistics/english-indices-of-deprivation-2010),
	Quality and outcomes framework (QOF) performance (as recorded by the Health and Social Care Information Centre: http://www.hscic.gov.uk/catalogue/PUB12262);

#### Reviewer agreement

Inter-rater agreement will be assessed through calculation of specificity and sensitivity for each of the four secondary reviewers relative to the primary reviewer using the phase 1 data. Agreement will be based on ratings for the latter 50 % of records in each case; with the first 50 % being regarded as principally a training set. Sensitivity and specificity rates of ≥75 % will be considered as acceptable, ≤60 % as requiring further calibration and 60–75 % as requiring input from the research team steering group to decide whether further calibration is required or not.

#### Study duration and timeline

The study started with literature review, planning, consultation, preparation and finalising this protocol. We have full ethical and R&D approvals, and data collection will begin soon. Data collection in all of the participating practices is estimated to last a maximum of 12–16 months. The study endpoint, including analyses and write-up is estimated to be December 2016 to March 2017.

#### Ethical and research and development approvals

Ethical approval for the study was obtained from NRES Committee North West-Lancaster on 23/03/15 (reference: 14/NW/1491). Approval was also obtained from the Confidential Advisory Group on 9/03/15 (reference: 14/CAG/1041).

We do not plan to seek individual patient consent for access to patient records. This is because the records will be reviewed by GPs who are currently, or have been within the last 12 months, employed by the NHS and no patient identifiable information will be collected or used in any part of the analysis or reporting of the study findings. In addition, GPs regularly perform and are expected to perform clinical audit as part of their normal work activities.

## Discussion

There are no reliable estimates of the incidence of MDOs in English general practice. Current estimates are based often on anecdotes, personal experience and a number of small studies conducted, for example in the UK (4,8) There are many challenges in measuring diagnostic error and MDOs and in developing and using good measurement instruments; and the ability to reduce diagnostic errors hinges on the ability to overcome measurement-related challenges [7, 24]. Aside from the lack of epidemiological accuracy, the inability to date to measure MDOs impedes the formulation of policies or interventions to address and reduce MDOs. Hence, this project is taking multiple precautions to ensure reliability and accuracy such as training and ongoing monitoring of reviewers to maximise calibration through a process of double coding, and setting sample sizes according to designated degrees of precision. As a result of this project, future research will have an available and a more reliable estimate of MDOs as a prerequisite to improvement, which the American Medical Association (AMA) has highlighted as a priority because the literature on improving safety in primary care is still evolving [2]. While a forthcoming Institute of Medicine report on diagnostic error is bound to shed more light on the importance of this area, an embedded implementation strategy will be needed to drive improvement or a quality cycle to alter strategies and measure for change [25]. Some have advocated the need for outcome measures in patient safety [26], and a methodology to estimate more reliable levels of MDOs will be a key step in that direction. Measureable outcomes must be used alongside training, policy, culture and feedback of outcomes data in line with other established models of patient safety [27, 28]. In addition to providing foundational data on diagnostic errors, findings from this study could be used also for educational purposes in terms of targeted training of both current and future clinicians.

## Additional files

Additional file 1: 
**Project introductory letter.** (DOCX 70 kb) Additional file 2: 
**Information sheet.** (DOCX 54 kb) Additional file 3: 
**Consent form.** (DOC 49 kb) Additional file 4: 
**Data extraction form.** (DOCX 47 kb) Additional file 5: 
**Practice and clinician demographic form.** (DOCX 31 kb) Additional file 6: 
**Coding manual.** (DOCX 50 kb)

## Abbreviations

AMA: American Medical Association
CCG: Clinical Commissioning Group
DE: diagnostic error
DEF: data extraction form
GM: Greater Manchester
GMC: General Medical Council
GMS: General Medical Services
Greater Manchester PSTRC: NIHR Greater Manchester Primary Care Patient Safety Translational Research Centre
GP: general practitioner
ICC: intra-cluster correlation coefficient
IMD: Index of Multiple Deprivation
IRR: inter-rater reliability
MDO: missed diagnostic opportunity
NHS: National Health Service
NIHR: National Institute of Health Research
NRES: National Research Ethics Service
PMS: Personal Medical Services
R&D: Research and Design
QOF: quality and outcomes framework
UK: United Kingdom
US: United States

## Footnotes

1 http://www.random.org/integer-sets/
