# Erratum to: Missed diagnostic opportunities and English general practice: a study to determine their incidence, confounding and contributing factors and potential impact on patients through retrospective review of electronic medical records

**DOI:** 10.1186/s13012-015-0314-1

**Published:** 2015-08-29

**Authors:** Sudeh Cheraghi-Sohi, Hardeep Singh, David Reeves, Jill Stocks, Morris Rebecca, Aneez Esmail, Stephen Campbell, Carl de Wet

**Affiliations:** NIHR Greater Manchester Primary Care Patient Safety Translational Research Centre, University of Manchester, 7th Floor: Williamson Building, Manchester, M13 9PL UK; Centre for Primary Care: Institute of Population Health, University of Manchester, 7th Floor: Williamson Building, Manchester, M13 9PL UK; Houston Veterans Affairs Centre for Innovations in Quality, Effectiveness and Safety, Michael E. DeBakey Veterans Affairs Medical Centre and Baylor College of Medicine, 2002 Holcombe Blvd. 152, Houston, 77030, 713.794.8601 TX USA; Centre for Research and Action in Public Health (CeRAPH), University of Canberra, Building 22, Floor B, University Drive, Bruce, 2617 ACT Australia; School of Medicine, Gold Coast Campus, Griffith University, Queensland, Australia

The original version of the article [1] unfortunately contained three mistakes. The statement for Hardeep Singh regarding VA funding was incorrectly listed in the ‘Competing Interests’ section. The correct location for this text is the ‘Acknowledgements’ section. The ‘Competing Interests’ section was also missing the text “All authors confirm that they have no competing interests”. Finally, the formatting for citation 7 was incorrectly displayed as Hardeep S. The correct formatting is Singh H.
