# Supplementary material for: Missed diagnostic opportunities and English general practice: a study to determine their incidence, confounding and contributing factors and potential impact on patients through retrospective review of electronic medical records
Source: Implement Sci. 2015 Jul 29;10:105. doi: 10.1186/s13012-015-0296-z (PMC4518650; doi:10.1186/s13012-015-0296-z)
Supplement: Additional file 2: — Information sheet. (DOCX 54 kb) [file 13012_2015_296_MOESM2_ESM.docx]

**Missed Diagnostic Opportunities and English general practice**

**Introduction**

You are being invited to take part in a study to investigate ‘missed diagnostic opportunities’ in English General Practice. The concept of a missed diagnostic opportunity essentially implies that something different could have been done to make the correct diagnosis earlier or that the diagnosis was incorrect. We are interested in looking at patient records to explore the processes that contribute to such missed diagnostic opportunities as well as any resulting impact(s).

Please take time to read the following information carefully. If there is anything you are not clear on or you would like to receive more information, please feel free to contact me Dr Rebecca Morris on 0161 275 0748 or [rebecca.morris@manchester.ac.uk](mailto:rebecca.morris@manchester.ac.uk).

Take time to decide whether or not you wish to take part.

Thank you for reading this information sheet.

**What is the aim of the research?**

There are three aims:

1. To determine the incidence of MDOs in English general practice
2. To identify the confounding and contributing factors that lead to MDOs.
3. To determine the impact, or potential impact of the MDOs detected in the study

**Why have I been asked to participate?**

Your practice is being asked to participate as it is an English general practice which meets the inclusion and sampling criteria (in terms of list size and level of deprivation) for our study.

**What would I be asked to do if I took part?**

If your practice agrees to participate you will not be asked to do anything directly, but simply to provide access and space for our practice level study team (an administrator plus two GP reviewers) to conduct the study. Specifically, we will provide a trained administrator to locate the necessary 100 patient records (which meet the inclusion criteria) from your system. Once this list of 100 patient records has been generated and stored on-site by the administrator, two GP reviewers will visit the practice on separate occasions (minimising disruption and space required) and each will independently work through the list generated by our administrator. The reviewers will be using a structured data collection tool and will not be collecting any patient identifiable information other than the patients’ age and gender. The reviewers will specifically be looking at information relating to the documented evidence within the electronic health record in order to ascertain the underlying diagnostic process for the selected records. We anticipate that the study will require approximately 5 days of access for the practice level study team. We are able to compensate you £1000 for your practice’s participation.

**Do I have to take part?**

No your practice and you do not. Your participation is entirely voluntary.

**What are the possible advantages of taking part**

We are essentially performing a structured audit of a sample of your patient records. During this process the reviewers may uncover potential issues which can fed back to you and your team for discussion at a practice meetings. We will feedback your practice-level results as soon as they are available. You may then wish to act upon the findings. We will also provide an aggregated summary of the findings.

**What are the possible disadvantages and risks of taking part?**

None. We will not be extracting any patient identifiable information other than age and gender (See below for precautions to ensure your data is protected). Finally, we are not asking you to devote any practice resources, except for our team to have the necessary space and access to your electronic patient record system.

**Confidentiality**

All practice level information extracted by the study will be kept strictly confidential. All data will be pseudo-anonymised when collected using an alpha-numeric system and upon completion of data analysis the link between practice level codes and data will be destroyed ensuring anonymity. The pseudo-anonymised data will be recorded on secure encrypted laptops and uploaded securely to the University’s secure server and deleted from the portable device upon University premises. All data will be accessed and analysed solely by named members of the research team. Data published will be at an aggregated level and individual practices and/or patients will not be identifiable.

**Can we withdraw?**

Once we have collected and analysed the data we will not be able identify your practice specifically therefore withdrawal will not be possible.

**What will happen to the results of the research study?**

The results may be presented at conferences and will be published in professional and academic journals.

**Who is organising, governing and funding the research?**

This research is being funded by National Institute of Health Research. This study is part of a larger programme of work that is investigating how patient safety can be improved in primary care.

The study that you are being asked to take part in is being led by Dr Rebecca Morris, Professor Stephen Campbell and Professor Aneez Esmail.

As a result data collected for this study may be accessed by individuals from the University of Manchester, regulatory authorities or NHS Trust for auditing and monitoring of the study. Access will be as per the terms of the NHE ethical and Confidential Advisory Group (CAG) approval and all individuals will have a duty of confidentiality to those taking part in the study.

**Who has reviewed the study?**

This study has been peer reviewed internally and externally as well as by an NHS Research Ethics Committee. Ref: 14/NW/1491

The study has also gained section 251 CAG approval from which provides a legal basis for the study team to have access to your records.

Section 251 was established to enable the common law duty of confidentiality to be overridden to enable disclosure of confidential patient information for medical purposes, where it was not possible to use anonymised information and where seeking consent was not practical, having regard to the cost and technology available. - See more at: http://www.hra.nhs.uk/about-the-hra/our-committees/section-251/what-is-section-251/#sthash.DU9yOCgt.dpuf

**What happens next?**

If you decide to take part in the study please complete the enclosed Expression of Interest Form (Version 1.0, 05/09/2014) and return it using the enclosed freepost envelope. A member of the research team will contact you thereafter to answer any questions and make further arrangements. If you do not wish to take part in the study you do not need to do anything.

**What if there is a problem?**

If you have a concern about any aspect of this study, you should ask to speak to the researchers who will do their best to answer your questions. If they are unable to resolve your concern or you wish to make a complaint regarding the study, please contact a University Research Practice and Governance Co-ordinator on 0161 275 7583 or 0161 275 8093 or by email to [research.complaints@manchester.ac.uk](mailto:research.complaints@manchester.ac.uk).

**Where can I get more information?**

If you have any questions about any aspect of the study please contact me or a member of the research team using the contact details below.

Dr Rebecca Morris

Centre for Primary Care

NIHR Greater Manchester Primary Care Patient Safety Translational Research Centre

7th Floor Williamson Building, University of Manchester, Williamson Building, Oxford Road, Manchester, M13 9PL

Email: [rebecca.morris@manchester.ac.uk](mailto:rebecca.morris@manchester.ac.uk).

Telephone: +44(0) 161 275 0748

Rahul Alam

Centre for Primary Care

NIHR Greater Manchester Primary Care Patient Safety Translational Research Centre

7th Floor Williamson Building, University of Manchester, Williamson Building, Oxford Road, Manchester, M13 9PL

Email: [Rahul.Alam@Manchester.ac.uk](mailto:Rahul.Alam@Manchester.ac.uk)

Telephone: +44(0)161 275 0747
