# Supplementary material for: Missed diagnostic opportunities and English general practice: a study to determine their incidence, confounding and contributing factors and potential impact on patients through retrospective review of electronic medical records
Source: Implement Sci. 2015 Jul 29;10:105. doi: 10.1186/s13012-015-0296-z (PMC4518650; doi:10.1186/s13012-015-0296-z)
Supplement: Additional file 3: — Consent form. (DOC 49 kb) [file 13012_2015_296_MOESM3_ESM.doc]

**
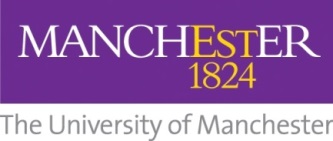
**

# Consent formv1 04/09/2014

**An invitation to your Practice to participate in:** **Missed Diagnostic Opportunities in**

**English general practice**

**If on reading the participant information sheet (v1 05/10/14) you are happy to participate please complete and sign the consent form below**

Anonymised practice ID:…………….

|  | Please Initial Box |
| --- | --- |
| 1. I confirm that I have read the attached information sheet (**v1 05/10/14**) on the above project and have had the opportunity to consider the information, ask any questions and have had these questions answered satisfactorily and am happy to proceed. |  |
| 2. We agree to allow the study team to have access to our patient records for the specific stated purposes of the above study. |  |
| 3. We understand that we are free to withdraw from this research should the aims of the study change. |  |
| 4. We understand that once the data has been collected and analysed we will not be able to withdraw as our data will not be identifiable. |  |

This practice agrees to take part in the above study (please circle as appropriate).

Yes / No

Name:……..…………………………………………….………………….

Role:……………………………………………………………………………

Signed: ……………………………………………………………..

General Practice name: ……..……………………………………………..

Study contact telephone number: ………………………………………………..

Study contact email address:………………………………………………………

Computer system (e.g. EMIS LV)…………………………………………
