# Supplementary material for: Missed diagnostic opportunities and English general practice: a study to determine their incidence, confounding and contributing factors and potential impact on patients through retrospective review of electronic medical records
Source: Implement Sci. 2015 Jul 29;10:105. doi: 10.1186/s13012-015-0296-z (PMC4518650; doi:10.1186/s13012-015-0296-z)
Supplement: Additional file 4: — Data extraction form. (DOCX 47 kb) [file 13012_2015_296_MOESM4_ESM.docx]

**Step one: Gather information about the index consultation**

**Unique codes for general practices, reviewers and records**

| Index Consultation code |  | Practice code |  | Reviewer code(s) | | | |
| --- | --- | --- | --- | --- | --- | --- | --- |
|  |  |  |  | R1: | R2: | R3: | SGR |

**Index consultation information**

| Date |  |  | |  |  |  |  |  |  |  |
| --- | --- | --- | --- | --- | --- | --- | --- | --- | --- | --- |
| Diagnostician ID |  |  | |  |  |  |  |  |  |  |
| Time |  | < am tea | | < lunch | < pm tea | < 18:00 | OOH |  |  |  |
| Place |  | Home visit | | Practice - acute | Practice - normal |  |  |  |  |  |
|  |  |  |  | | | | | |  |  |

**Patient characteristics**

| Male | Female |  | Age (years) |  |
| --- | --- | --- | --- | --- |

| **Repeat medication items** |  |
| --- | --- |

| **Long-term conditions** | | |  |  | |  |  |  |
| --- | --- | --- | --- | --- | --- | --- | --- | --- |
| Name (list) |  |  | | |  |  |  |  |
| Additional |  |  | | |  |  |  |  |
| Additional |  |  | | |  |  |  |  |
| Additional |  |  | | |  |  |  |  |

**Step two: Record the diagnoses in the index consultation**

| **Are there one or more diagnoses?** | Yes | No |
| --- | --- | --- |

**Document the presenting symptoms and diagnosis/es recorded in the index consultation**

|  |  | Dx1 | Dx2 | Dx3 |
| --- | --- | --- | --- | --- |
| Symptoms and Diagnosis (words) |  |  |  |  |

**Can diagnoses be inferred (if yes=Dx4, Dx5) AND/OR was a diagnosis provided by another clinician (yes=Dx6)?**

|  | |  | Dx4 | Dx5 | Dx6 |
| --- | --- | --- | --- | --- | --- |
| Diagnosis (words) |  | |  |  |  |

**Step three: Gather evidence relevant to diagnosis/es**

**Tick one box (✓) next to each ‘trigger’ (T) each time you find it in the record.**

| **Trigger (T)**  (A ‘prompt’ that may indicate a diagnostic error) | **Total** |  | | | | | | | |  |  | | | | | | | | | | | **Total** |
| --- | --- | --- | --- | --- | --- | --- | --- | --- | --- | --- | --- | --- | --- | --- | --- | --- | --- | --- | --- | --- | --- | --- |
|  |  |  |  |  |  |  |  |  |  |  |  |  |  |  |  |  |  |  |  |  |  |  |
| T1. Consultations within ± 1 calendar month |  |  |  |  |  |  |  |  |  |  |  |  |  |  |  |  |  |  |  |  |  |  |
|  | | | | | | | | | | | | | | | | | | | | | | |
|  |  | 3 months **before** the target consultation under review (T2-T6) | | | | | | | |  | 9 months **after** the target consultation under review (T2-T6) | | | | | | | | | | |  |
|  |  |  |  |  |  |  |  |  |  |  |  |  |  |  |  |  |  |  |  |  |  |  |
| T2. Referrals |  |  |  |  |  |  |  |  |  |  |  |  |  |  |  |  |  |  |  |  |  |  |
|  |  |  |  |  |  |  |  |  |  |  |  |  |  |  |  |  |  |  |  |  |  |  |
| T3. Hospital admissions |  |  |  |  |  |  |  |  |  |  |  |  |  |  |  |  |  |  |  |  |  |  |
|  |  |  |  |  |  |  |  |  |  |  |  |  |  |  |  |  |  |  |  |  |  |  |
| T4. GP out of hours attendance |  |  |  |  |  |  |  |  |  |  |  |  |  |  |  |  |  |  |  |  |  |  |
|  |  |  |  |  |  |  |  |  |  |  |  |  |  |  |  |  |  |  |  |  |  |  |
| T5. A&E attendance |  |  |  |  |  |  |  |  |  |  |  |  |  |  |  |  |  |  |  |  |  |  |
|  |  |  |  |  |  |  |  |  |  |  |  |  |  |  |  |  |  |  |  |  |  |  |
| T6. Imaging requests |  |  |  |  |  |  |  |  |  |  |  |  |  |  |  |  |  |  |  |  |  |  |
|  |  |  |  |  |  |  |  |  |  |  |  |  |  |  |  |  |  |  |  |  |  |  |

**Consider each question in relation to Dx1 to Dx6 and rate your level of agreement, if relevant**

|  |  |  | Dx1 | Dx2 | Dx3 | Dx4 | Dx5 | Dx6 |
| --- | --- | --- | --- | --- | --- | --- | --- | --- |
| Earlier encounters | Q1. Do earlier encounters (history, examination, differential diagnoses) suggest an alternate index diagnosis that was not considered? |  |  |  |  |  |  |  |
|  |  |  |  |  |  |  |  |  |
| Index consultation | Q2. Were available diagnostic testing data suggesting an alternate diagnosis not considered or misinterpreted? |  |  |  |  |  |  |  |
|  |  |  |  |  |  |  |  |  |
|  | Q3. Were there any ‘red flag’ symptoms or signs that were not acted on? |  |  |  |  |  |  |  |
|  |  |  |  |  |  |  |  |  |
|  | Q4. Should any other clinical information in the index consultation have prompted additional evaluation (referral, investigation, follow-up appointment)? |  |  |  |  |  |  |  |
|  |  |  |  |  |  |  |  |  |
|  | Q5. Was the ‘final’ diagnosis an evolution of the index diagnosis? |  |  |  |  |  |  |  |
|  |  |  |  |  |  |  |  |  |
|  | Q6. Was the clinical presentation at the index consultation atypical? |  |  |  |  |  |  |  |
|  |  |  |  |  |  |  |  |  |
| Subsequent encounters | Q7. Do subsequent encounters (OOH, A&E, specialist clinics, practice consultations) suggest missed diagnostic opportunities? |  |  |  |  |  |  |  |
|  |  |  |  |  |  |  |  |  |

Rating scale: 1=Strongly agree; 2=Agree; 3=Disagree; 4=Strongly disagree; 5=n/a

**Step four: Rate and describe diagnoses and missed diagnostic opportunities**

| **Rate the perceived accuracy of each index diagnosis** | | | | | | | |
| --- | --- | --- | --- | --- | --- | --- | --- |
|  |  | Dx1 | Dx2 | Dx3 | Dx4 | Dx5 | Dx6 |
| Reviewer rating |  |  |  |  |  |  |  |

Rating scale: 1=diagnosis accurate and sufficient evidence; 2=diagnosis accurate but insufficient evidence; 3=diagnosis accurate but incomplete and/or little or no evidence; 4=missed diagnostic opportunity (MDO) possible; 5=MDO likely; 6=MDO certain

**Indicate your degree of confidence in the rating of the accuracy of the index diagnoses**

|  |  | Dx1 | Dx2 | Dx3 | Dx4 | Dx5 | Dx6 |
| --- | --- | --- | --- | --- | --- | --- | --- |
| Reviewer rating |  |  |  |  |  |  |  |

Rating scale: 1=Very confident; 2=confident; 3=somewhat confident; 4=a little uncertain; 5=uncertain; 6=very uncertain

| **Rate the impact of missed diagnostic opportunities on patients** | | | | | | | |
| --- | --- | --- | --- | --- | --- | --- | --- |
|  |  | Dx1 | Dx2 | Dx3 | Dx4 | Dx5 | Dx6 |
| Reviewer rating |  |  |  |  |  |  |  |

1=No harm – includes ‘near misses’; 2=Mild harm: inconvenience, further follow-up or investigations; 3=Moderate harm: self-limiting physical or psychological distress

4=Severe harm: prolonged or permanent impact on patient - including preventable admissions; 5=Unclear

| **What were the main contributing factors to the missed diagnostic opportunities?** | | | | | | | |
| --- | --- | --- | --- | --- | --- | --- | --- |
|  |  | Dx1 | Dx2 | Dx3 | Dx4 | Dx5 | Dx6 |
| Reviewer rating |  |  |  |  |  |  |  |

Contributing factors: 1= Patient-practitioner clinical encounter 2= Performance and/or interpretation of diagnostic tests, 3 =Follow-up and tracking of diagnostic information, 4=Subspecialty and referral-related, 5=Patient-specific processes, 6=Unclear

| **Summarize each missed diagnostic opportunity**  DX1 –  DX2-  DX3-  DX4-  DX5-  DX6 - |
| --- |

| **List all confounding factors for each missed diagnostic opportunity**  DX1 –  DX2-  DX3-  DX4-  DX5-  DX-6- |
| --- |

| **Reviewer comments for each missed diagnostic opportunity**  DX1 –  DX2-  DX3-  DX4-  DX5-  DX6- |
| --- |
