# Supplementary material for: Missed diagnostic opportunities and English general practice: a study to determine their incidence, confounding and contributing factors and potential impact on patients through retrospective review of electronic medical records
Source: Implement Sci. 2015 Jul 29;10:105. doi: 10.1186/s13012-015-0296-z (PMC4518650; doi:10.1186/s13012-015-0296-z)
Supplement: Additional file 5: — Practice and clinician demographic form. (DOCX 31 kb) [file 13012_2015_296_MOESM5_ESM.docx]

**Practice demographics form**

###### Section 1: PRACTICE STAFF

###### Name of Practice:

- 1. How many staff currently work in the practice:

Number of GPs ……………..

Number of Nurse Practitioners/prescribers………………..

Number of Practice Nurses.......…....

Number of Administrative Staff (including practice manager)………………

Other……………

**Date of Index Consultations**

**Date of Emis Web or other electronic record installation at this practice:**

**Index Consultation dates for this practice:**

**Week 1**

**Week 2**

**Week 3**

**Week 4**

1.2. For each **GP and nurse prescriber** working at the practice please complete the following:

| ID | Gender | Type of GP (permanent, locum, other) | Number of clinic sessions/week | If GP specialist, in which area? | Years fully qualified: (GMC registration if GP) | Present at time of Index Consultation (y/n) |
| --- | --- | --- | --- | --- | --- | --- |
|  |  |  |  |  |  |  |
|  |  |  |  |  |  |  |
|  |  |  |  |  |  |  |
|  |  |  |  |  |  |  |
|  |  |  |  |  |  |  |
|  |  |  |  |  |  |  |
|  |  |  |  |  |  |  |
|  |  |  |  |  |  |  |
|  |  |  |  |  |  |  |
|  |  |  |  |  |  |  |
|  |  |  |  |  |  |  |
|  |  |  |  |  |  |  |
|  |  |  |  |  |  |  |
|  |  |  |  |  |  |  |
|  |  |  |  |  |  |  |
|  |  |  |  |  |  |  |
|  |  |  |  |  |  |  |
|  |  |  |  |  |  |  |
|  |  |  |  |  |  |  |

**Were there any other GPs or Nurse Prescribers present at the time of the Index Consultation dates not listed above?**

**(*please continue on a separate sheet if necessary*)**

| **ID** | **Gender** | **Type of GP (permanent, locum, other)** | **Number of clinic sessions/week** | **If GP specialist, in which area?** | **Years fully qualified: (GMC registration if GP)** | **Present at time of Index Consultation (y/n)** |
| --- | --- | --- | --- | --- | --- | --- |
|  |  |  |  |  |  |  |
|  |  |  |  |  |  |  |
|  |  |  |  |  |  |  |
|  |  |  |  |  |  |  |

###### Section 2: Organisation

2.1. Are you a training practice? Yes 📺 No 📺

2.2. What type of contract does the practice hold (GMS, PMS)?...................................

2.3. Do you have any branch surgeries? Yes 📺 No 📺

2.4. What computer system does the practice use (e.g. EMIS, Vision,etc) ………………………..

2.5 When was your computer system installed?

2.6. What is the current list size of the practice? ….......................

2.7. What are the practice’s normal opening hours, including extended hours?

Mon ….………

Tues…….……

Wed….………

Thur ….………

Fri ……….…

Sat ………….

Sun………………
