# Supplementary material for: Missed diagnostic opportunities and English general practice: a study to determine their incidence, confounding and contributing factors and potential impact on patients through retrospective review of electronic medical records
Source: Implement Sci. 2015 Jul 29;10:105. doi: 10.1186/s13012-015-0296-z (PMC4518650; doi:10.1186/s13012-015-0296-z)
Supplement: Additional file 6: — Coding manual. (DOCX 50 kb) [file 13012_2015_296_MOESM6_ESM.docx]

**Coding Manual**

| **Step one: Gather information about the index consultation** |
| --- |

**Unique codes for anonymisation:**

*Practice code*

Each general practice in the UK already has a unique five digit numerical code. To help protect the anonymity of practices and the confidentiality of the data extraction forms (DEF), each practice will be assigned a further, unique practice code consisting of two letters. For example, a practice with the code of 12345 will be assigned a code of ‘AA’, the next practice ‘AB’ and so forth. This code should be entered into the space below ‘practice code’ on the electronic DEF. The two letter code is used to create the ‘index consultation code’ (below).

*Index Consultation code*

Every individual patient record in the study will have a unique identifier – the ‘index consultation code’ – assigned to it and this should be documented on the electronic DEF. The ‘index consultation code’’ is formed by combining the practice code (see above) with consecutive numbers consisting of three digits. For example, if the first practice has been assigned a code of ‘AA’, the first record of the sample that will be reviewed in that practice will be assigned a code of ‘AA001’. The next record’s unique code will be ‘AA002’, and so forth.

*Reviewer code*

The reviewer code is the initials of the clinician conducting the review. For example, if Jane Smith performed the review, she would enter ‘JS’ in the space below ‘reviewer code’. Each DEF should have at least one reviewer code. If a second reviewer is involved in the review in any capacity – whether for advice, to ‘check’ the ratings of the first reviewer or to conduct a full review, she/he should enter their initials in the second space below ‘reviewer code’.

NOTE: The list of participating practices, their unique codes, study codes and details will only be accessible to researchers (throughout the study), the clinical reviewers and the administration support team (during the data collection period). Individual patient IDs linked to patient names and details will NOT leave participating practice premises. The file will be stored in a secure folder on the practice server.

**Patient characteristics**

*Gender*

Select the appropriate option, ‘male’ or ‘female,’ to indicate the patient’s gender.

*Age*

Enter the age of the patient (in years) at the time of the index consultation. For example, a patient’s age is displayed as ‘88’ when you open her record to prepare the DEF on the 1^st^ July 2014. However, her date of birth is the 1^st^ of June 2014 and the index consultation was on the 1^st^ of August 2013. In this example, the correct age to enter in the space next to ‘age’ on the DEF would be ‘87’.

**Repeat medication items**

Enter the total number of medication items as they are listed on the day the DEF is being prepared for review. Every repeat item should be counted, irrespective of type, e.g. oral, injectable or topical. However, repeat medication items that have *not been issued* at any time during the preceding twelve months (or longer) should not be included in the count.

NOTE – the number of repeat medication items may have been different on the day of the index consultation/diagnosis, but is not feasible to extract this information direct using the available software packages.

Some medication items are prescribed more than once, but with different dosages. In these instances the medication should be counted only once, as demonstrated through the following three examples:

- Example 1: A patient with atrial fibrillation is prescribed warfarin 5mg, warfarin 3mg and warfarin 1 mg tablets as repeat medication items. All three items should be counted as one repeat medication item.
- Example 2: A patient with heart failure is prescribed Bisoprolol 2.5mg and Bisoprolol 5 mg tablets as repeat medication items. All three items should be counted as one repeat medication item.
- Example 3: A patient with asthma is prescribed a Salbutamol inhaler and a Seretide inhaler. This should be counted as two repeat medication items.

**Long-term conditions**

Enter the total number of long-term conditions according to the guidance and list of 25 items below. If there are no conditions, enter a ‘0’ in the space provided.

EXCLUDE those long-term conditions indicated as minor.

The following long-term conditions and medical problems should be included on the DEF. Each bulleted item on the list should be counted once only. For example, a patient with Gastro-oesophageal reflux disease (GORD) and oesophagitis should only have these counted as *one* long-term condition. The codes for each condition that will be used when preparing the data for analysis are shown in brackets for interest.

- COPD (C01)
- Asthma (C02)
- Atrial fibrillation or atrial flutter or any other ‘permanent’ or recurrent arythmia (C03)
- Heart failure (C04)
- Ischaemic heart disease or angina (any type) or coronary heart disease or myocardial infarction (C05)
- Hypertension of any type (C06)
- Previous stroke/TIA/CVA (C07)
- Hypothyroidism or hyperthyroidism (C08)
- Diabetes mellitus, whether diet controlled, type 1 or type 2 (C09)
- Gastro-oesophageal reflux disease (GORD) or oesophagitis or Barret’s oesophagus or hiatus hernia (C10)
- Dyspepsia or peptic ulcer disease or duodenal ulcer or gastritis or *Helicobacter pylori* or gastric pain (C11)
- Inflammatory bowel disease, including crohn’s disease and ulcerative colitis (C12)
- Diverticular disease / diverticulosis (C13)
- Irritable bowel syndrome (C14)
- Any inflammatory arthropathy, including rheumatoid arthritis (C15)
- Any non-inflammatory arthritis, including osteo-arthritis but excluding chronic pain syndromes including Not Otherwise Specified (NOS) (C16)
- Chronic pain or back pain or sciatica or fibromyalgia or ME (C17)
- Dementia (C18)
- Parkinson’s disease or any other disorder of movement (C19)
- Any mental health problem, including depression, bipolar disorder, schizophrenia and anxiety (C20)
- Substance abuse or dependence of any type (21)
- Chronic kidney disease (any stage) or related renal pathology with a chronic and/or recurrent nature, e.g. recurrent UTIs, recurrent stones or polyps (C22)
- Cancer (C23): specify the primary site (if known and coded). If the patient has more than one kind of cancer, each one should be counted separately. For example, a patient with skin cancer (first long-term condition) subsequently and unrelated to this develops colon cancer (second long-term condition). However, metastatic disease and recurrent cancer should not be counted as additional conditions.
- Sensory impairment, including deafness and impaired sight. This item includes: glaucoma, cataracts, macular degeneration and all types of retinopathy. (C24)
- Any chronic and/or recurrent complications resulting from surgical procedures such as fistulas, adhesions or paraplegia. (C25) However, surgical procedures should not be counted as long-term conditions. They may be valuable indicators of other multimorbidities which should be included though. For example, a clinical entry in the record about a hip replacement may alert you to the presence of osteo-arthritis.
- Anaemic (any type) or any vitamin deficiency (C26)
- Osteoporosis (C27)
- Chronic Prostate and urinary tract symptoms including incontinence (C28).
- Migraine, chronic headaches or any other type of chronic or recurring headache (C29)

In addition to the list above, any other condition that meets all of the following criteria should also be counted:

- It is long-term and/or recurrent in nature; and
- It has a potential adverse impact on patients’ health and wellbeing; and
- It has been coded in the medical record.

In these instances, *record ‘other’* (C30) in the spaces provided on the DEF.

There is no maximum limit to the number of long-term conditions that can be entered. If there are more long-term conditions than the 20 spaced provided on the DEF, enter them in the ‘reviewer comments’ section.

**Consultations**

*Diagnostician*

Enter the pre-assigned ID for the diagnostician indicated in the index consultation.

*Type of consultation*

Practices offer a range of different appointments to their patients. If it is clear from the record or the practices’ dairies, then the type of consultation should be indicated on the DEF. The options are:

- Home visit;
- Practice

*Date of consultation*

Enter the date of the index consultation in the box using the format dd/mmm/yy.

NOTE – The date should be coded as a day of the week when the DEF data are prepared for analysis.

*Time of consultation*

- Indicate the time of the consultation by marking one of the appropriate boxes. OOH (out of hours) – any time between 18:00 and 08:00 am during the week or any time during the weekend.

NOTE – The OOH option is included on the DEF to differentiate between ‘normal’ working hour and extended hours consultations.

| **Step Two. Record the diagnoses in the index consultation** |
| --- |

Step two is essential to the review process. If there are no new recorded diagnoses for an index consultation (record), the review effectively stops at that point, as it is then impossible to determine whether there had been a missed diagnostic opportunity. It is also essential that the index diagnosis/es are documented correctly on the DEF – this does not imply that the diagnosis/es were necessarily correct, but the DEF should accurately reflect the record.

There are three possible types of diagnoses a reviewer should consider:

1. Those that were recorded by the diagnostician i.e. new diagnoses (DxN);
2. Diagnoses that can be inferred from the record (DxI) and these may be in two forms:
   - ‘Diagnosis by action:’ Here the GP reviewers infers that a diagnosis has been made by the clinician during the index consultation by using both the recorded symptom and ensuing actions e.g. treatments etc.
   - ‘Diagnosis inferred by reviewer’: Here the GP reviewer infers from the recorded index consultation information that a diagnosis should have or could have been made by the clinician in the index consultation but was not made/recorded.
3. Diagnoses that were made before the index consultation by another clinician but that comes to light during the index consultation (DxC)

NOTE – the main focus of this study is the first type of diagnoses (DxN). Capturing data about all types of diagnoses may be informative about related issues, for example for determining how likely clinicians are to record a diagnosis.

**Is a diagnosis recorded?**

Indicate whether a diagnosis or diagnoses are recorded in the index consultation. Depending on the patient record and practice software, the diagnoses may be found in the clinical entry or alternatively have been entered as a clinical code.

If a diagnosis/es have been recorded, each one and the associated presenting symptoms should be documented consecutively on the form as DXN1, DXN2 etc and DXI, DxC etc.

**Can a diagnosis be inferred?**

In some instances, a diagnosis may not be recorded in the data entry for the index consultation, but a reviewer is able to infer a diagnosis from the recorded information in the record. This option is completely left to the discretion of the reviewer, who is encouraged to draw on her clinical acumen to make a decision whether the clinical entry offers sufficient information so that a diagnosis can be inferred with reasonable certainty. It is important that the reviewer records the inferred diagnosis as they imagine the diagnostician perceived it at the time of the index consultation. The reviewer should not enter an inferred diagnosis after reviewing the rest of the record.

Inferred diagnoses and their associated symptoms should be coded as ‘DxI’ and. In the unlikely event that there are more than one inferred diagnoses, this should be recorded in ‘reviewer comments’.

This section of the form can be explained further through three practical examples.

*Example one*

The clinical entry for the index consultation was: ‘...One day of burning rash affecting left jaw only. Blisters, macules. Treat with oral acyclovir and review if needed...’ In this example there was no recorded diagnosis, but a reviewer inferred that the diagnostician was considering shingles. The reviewer therefore coded ‘shingles’ as ‘DxI’ on the DEF.

*Example two*

The clinical entry for the index consultation was: ‘...three days productive green sputum, occasional rhonchi and wheeze – treat Amoxicillin’. In this example, the reviewer inferred that the diagnosis was ‘chest infection’ and codes this as ‘DxI’ on the DEF.

*Example three*

During a consultation with an elderly patient, the GP recorded a BP reading of 178/95, commenced anti-hypertensive treatment and arranged follow-up review with the practice nurse. The inferred diagnosis in this example was recorded as ‘uncontrolled hypertension’ by the reviewer.

**Was there a diagnosis from another clinician or setting that becomes apparent during the index consultation?**

In a small number of instances a diagnosis is made by a clinician (from the practice or in another health care setting) before the index consultation. However, the diagnosis only becomes ‘relevant’ during the index consultation. This type of diagnosis and associated symptoms should be coded as ‘DxC’. Only a *new* diagnosis should be coded as ‘DxC’. In the unlikely event that there is more than one of this type of diagnosis, this should be recorded in ‘reviewer comments’. This section of the DEF is further illustrated through three practical examples (below).

*Example one*

A patient attended secondary care for excision of a skin lesion. She attends the practice a few weeks later to enquire about the histology report. Her GP checks the hospital database and informs her that the result is ‘Bowen’s disease’. In this example a diagnosis is recorded in the record and should be coded as ‘DxC’. The GP (the diagnostician during the index consultation) did not make the diagnosis, but simply informed the patient of it.

*Example two*

A patient attends the orthopaedic outpatient clinic because of severe hip pain. They request x-rays that confirm osteoarthritis and advise her to return to her GP for additional analgesia. She returns to the practice (the index consultation) and informs the GP about the outcome of the clinic appointment and result of the x-ray. However, ‘osteoarthritis’ has previously been recorded in her record. In this example, there is not a ‘new’ diagnosis recorded in the index consultation and ‘osteoarthritis’ would not be coded as ‘dx6’.

*Example three*

A patient attends the practice nurse clinic for a general review. During the next few visits she records abnormally high blood pressure readings and make an appointment with a GP (the index consultation). The GP places the patient on the ‘hypertension’ register and commence antihypertensive treatment. In this example, ‘hypertension’ should be coded as ‘dx6’.

| **Step three. Gather evidence relevant to the diagnosis/es** |
| --- |

The main objective for reviewers in step three is to systematically gather enough evidence about the diagnoses in the index consultation so that they are able to make a professional judgment about the accuracy of each diagnosis and to decide whether there had been missed diagnostic opportunities. This can be achieved by performing two related tasks: (i) searching for the presence of six predefined triggers that could be indicative of the presence of missed opportunities and (ii) by considering a number of questions that were specifically developed for conducting primary care record reviews.

**Triggers**

Triggers are ‘prompts’ that help reviewers navigate (e.g. search) the different sections of a medical record in a focused, structured and active manner. Any primary care record – whether electronic or paper-based – has a maximum of only *five main sections*. They are listed in Box 1, with examples of previously defined and validated triggers that are commonly used when the Trigger Review Method (TRM) is applied in general practice settings.^^[[1]](#footnote-1)^^

*Box 1. The sections of any primary care record with examples of pre-defined triggers*

| **Section** | **Trigger** |
| --- | --- |
|  |  |
| Clinical encounters | - ≥3 clinical encounters in any given 7 day period |
|  |  |
| Medication | - ‘Repeat’ medication item discontinued |
|  | - Optional triggers: - Acute prescription of NSAIDs - Acute prescription of opiates |
|  |  |
| Clinical codes | - A clinical read code for an adverse drug event and/or allergy |
|  | - Any new ‘high priority’ clinical code |
|  |  |
| Correspondence | - Out-of-hours (OOH) and/or Accident & Emergency contacts |
|  | - Emergency hospital admissions |
|  |  |
| Investigations | - Haemoglobin ≤ 10,0 g/dl. |
|  | - Optional triggers: - INR > 5 or < 1.7; - AST/ALT > 100 IU/L - eGFR deteriorates >5 |

The DEF has a list of six triggers that were specifically developed for this study. Reviewer should search every medical record for the presence of each of these triggers in turn. More specifically, they should search for the triggers in all five sections of the medical record, but only for the three month period *before* and the nine month period *after* the index consultation.

Reviewers should indicate (using the buttons) in the relevant box each time they detect a trigger. Importantly, they should then *read that section* of the record and *actively consider whether there is any evidence in it that may be* ***relevant* to the index consultation**. In addition to the essential, ‘triggered’ record contents, reviewers may also need to look at any other section of the record if they feel this may be relevant to help clarify the index consultation or provide additional evidence to help determine the accuracy of the index consultation diagnoses.

NOTE - It is unnecessary to routinely enter trigger ‘totals’, as this will automatically be done.

NOTE - The presence or absence of triggers does not necessarily imply that there were missed diagnostic opportunities.

**Trigger 1. Consultations**

The index consultation is the ‘starting point’ of the review and is **not** counted or indicated in the trigger section. Every other consultation (face to face, telephone etc) *within a 30 day period before and after the index consultation* is a ‘trigger’ and should be indicated with a cross in the relevant box. Reviewers should scan each of these consultations for evidence that may suggest a missed diagnostic opportunities and also consider the specific questions, especially Q1 and Q8. It may also be necessary and *appropriate to read the clinical entries of other consultations* within the twelve month period of review but outside the critical 60 day period, depending on the presence of the other triggers. However, this should not routinely be done and reviewers have to guard against being distracted by information that is not relevant to the index consultation.

Consultations that should be considered ‘triggers’ include:

1. Any face-to-face encounter between a patient and clinician, e.g.GP, GPST, GP Retainer etc;
2. Telephone discussions between a clinician and the patient; and
3. Home visits.

**Advice calls to or from secondary care or non-clinical practice team members phoning patients to arrange appointments or to inform patients of results are not ‘consultation’ triggers.**

**Trigger 2. Referrals**

Reviewers should scan referrals during the review period (three months before and nine months after the index consultation) for evidence of missed diagnostic opportunities. Referrals include any request to another health care agency that has been recorded in patients’ medical records, but exclude referrals specifically for imaging. One cross should be made in the appropriate box for each detected referral.

While it may be necessary and useful to review outpatient correspondence, this should not be done routinely and should not be ‘ticked’ as a referral.

**Trigger 3. Hospital admissions**

Reviewers should scan documentation relating to hospital admissions during the review period (three months before and nine months after the index consultation) for evidence of missed diagnostic opportunities. Any hospital admission should be considered to be a trigger, irrespective of its duration, whether it was elective or who made the decision to admit the patient.

**Trigger 4. Out-of-hours contacts**

The OOH trigger should only be ticked as being present if a face-to-face encounter between a patient and clinician had taken place, whether in a centre or the patient’s home. Initial or subsequent triage-related calls should not be included.

NOTE – the rationale for this decision is that some triage systems use non-clinicians to do a preliminary screen when patients phone.

**Trigger 5. Accident & Emergency attendances**

Reviewers should scan Accident & Emergency (A&E) correspondence relating to attendances during the review period (three months before and nine months after the index consultation) for evidence of missed diagnostic opportunities relating to the index consultation. A&E attendances as a result of patients being referred to be admitted to hospital should not be recorded as a trigger as they simply ‘pass through’ the department in these instances.

**Trigger 6. Imaging**

The imaging trigger is present if there is a record of any form of imaging being *requested or received* by the practice during the review period (three months before and nine months after the index consultation). Reviewers should evaluate the imagining results for evidence of missed diagnostic opportunities and also consider the specific questions, especially Q7, which deals with diagnostic testing data.

Imaging requests are most likely to be for x-rays, DEXA scans or ultrasound, although a minority of practices may have access to CT or MRI. If the imaging trigger is positive, a brief description of the type of imaging modality should be made in ‘reviewer comments’.

**Trigger 7. Laboratory and Pathology Tests**

The Laboratory and Pathology trigger is present if there is a record of any form of laboratory or pathology tests being *requested or received* by the practice during the review period (three months before and nine months after the index consultation). Reviewers should evaluate the test results for evidence of missed diagnostic opportunities and also and also consider the specific questions, especially Q3, which deals with diagnostic testing data. If the Laboratory and Pathology trigger is positive, a brief description of the type of tests should be made in ‘reviewer comments’.

**Specific questions**

Reviewers should actively consider eight specific questions while reviewing the record. The first question relates to clinical encounters in the three months before the index consultation, the next five questions are about the index consultation itself, while the final question considers the record contents of the nine months after the index consultation.

Each question/prompt should be answered by the reviewer by indicating their agreement using the rating scale provided on the DEF. The eight questions are listed and described below and summarized on the DEF.

| Q1. The content of earlier encounters **3 months prior to the index consultation** (history, examination, differential diagnoses) suggest an alternate index diagnosis that was not considered |
| --- |
|  |
| Q2. The content of (history, examination, differential diagnoses) suggests an alternate diagnosis that was not considered |
|  |
| Q3. Diagnostic testing data (labs, radiology, pathology etc) were suggestive of an alternate diagnosis which was not considered or misinterpreted |
|  |
| Q4. Alarm symptoms or ‘Red flag’ (i.e. features in the clinical presentation that are considered to predict serious disease) were not acted on |
|  |
| Q5. Clinical information present in the index consultation should have prompted additional evaluation (examination, referral, investigation, follow-up appointment) |
|  |
| Q6. The ‘final’ diagnosis was an evolution of the initial presumed index diagnosis |
|  |
| Q7. The clinical presentation at the index consultation was atypical given the final diagnosis |
|  |
| Q8. Subsequent encounters **9** **months since the index consultation** (OOH, A&E, specialist clinics, practice consultations) suggest missed diagnostic opportunities |

| **Step 4. Rate and describe diagnoses and missed diagnostic opportunities** |
| --- |

**Rate the accuracy of each diagnosis**

Consider all the evidence gathered during step 3 of the review before rating each diagnosis from the index consultation, using the supplied 6-point rating scale. Ratings of **1 to 3** imply that there were **no missed diagnostic opportunities**, e.g. diagnostic errors. Ratings **of 4 to 6** imply that there **were missed diagnostic opportunities** (diagnostic errors). Each point on the rating scale is described below and illustrated through a number of clinical examples.

*Diagnosis accurate and sufficient evidence (1)*

To be rated as ‘1’ an index diagnosis should have both the following characteristics: (i) it should be clear, specific and ‘complete’; and (ii) it should be confirmed by sufficient and documented evidence from the medical record. If a patient does not consult again because of the index problem, this is not to be considered as ‘evidence’ that the diagnosis was correct or that it had resolved.

- Example 1: The diagnosis in the index consultation is ‘epidermoid cyst of eyelid’. The patient is referred to an ophthalmologist who excises the lesion and histology confirms the diagnosis.
- Example 2: The diagnosis in the index consultation is ‘anal fissure’. Previous encounters provide additional clinical information (a history of constipation), a specialist examines the patient during an outpatient appointment and confirms the diagnosis, and the patient subsequently attends the practice with complete resolution of her symptoms.
- Example 3: The diagnosis in the index consultation is ‘tennis elbow’ (it could equally have been ‘lateral epicondylitis’). The patient is referred to the physiotherapy department who confirms the diagnosis. The patient receives a corticosteroid injection and returns a few weeks later with complete resolution of her symptoms.

*Diagnosis accurate but insufficient evidence (2)*

To be rated as ‘2’ an index diagnosis should have at least one of the following characteristics: (i) it should be clear and accurate but be less precise and/or specific compared with a rating of ‘1’; and (ii) there should be at least some documented evidence of its accuracy in the medical record.

- Example 1. The diagnosis in the index consultation is ‘lower back pain’. The patient is referred to the local physiotherapy department and they diagnose ‘sciatica’. In this example the index diagnosis is not precise enough to have been rated as ‘1’.
- Example 2. The diagnosis in the index consultation is ‘possible malignancy’. CT scan and biopsy results confirm a diagnosis of ‘colon cancer’. In this example the index diagnosis is not precise enough to have been rated as ‘1’.
- Example 3. A young patient presented with worsening epigastric pain after several episodes of alcohol intoxication. The index diagnosis was ‘alcohol related gastritis’. The patient was prescribed antacid treatment and advised on alcohol cessation. She did not have any further contact with medical services after this. In this example the index diagnosis is specific, clear and judged to be accurate, but there is not enough documented evidence to rate it as ‘1’.

*Diagnosis accurate but incomplete or little/no evidence (3)*

To be rated as ‘3’ an index diagnosis should have at least one of the following characteristics: (i) it should be related to the ‘final’ diagnosis but be less precise, clear and/or specific compared with ratings of ‘2’ and ‘1’; and/or (ii) there should be very little or no documented evidence of its accuracy in the medical record.

- Example 1. The diagnosis in the index consultation was ‘chesty cough’. The final diagnosis of ‘bronchiectasis’ was confirmed by a respiratory physician and CT scan result. In this example, the index diagnosis is actually only a symptom, but one that is related to the final diagnosis.

*Missed diagnostic opportunity possible (4)*

To be rated as ‘4’ an index diagnosis should have both of the following characteristics: (i) be different when compared with the final diagnosis, but the difference should be less than for ratings of ‘5’ or ‘6’; and (ii) there should be little or no documented, objective evidence in support of a diagnostic error. In other words, the judgment relied mainly on the reviewers’ professional interpretation.

- Example 1. The index diagnosis was ‘insomnia’. Further review of the records suggests the underlying, and arguably more important problem, was alcohol dependence. There was a potential missed diagnostic opportunity to identify this problem during the index consultation, although this can only be inferred.

*Missed diagnostic opportunity likely (5)*

To be rated as ‘5’ an index diagnosis should have both the following characteristics: (i) it should be substantially different from the final diagnosis; and (ii) there should be at least some documented evidence to support the presence of a missed diagnostic opportunity.

- Example 1. A patient presented with arm pain and an index diagnosis of ‘ulnar nerve entrapment’ was made. Subsequent physiotherapy review and x-ray report suggested a final diagnosis of ‘referred pain secondary to cervical spondylosis’. In this example, the index and final diagnosis is different, but relate to the same main anatomical system (musculoskeletal) *and* pathological process (mechanical impingement)

*Missed diagnostic opportunity certain (6)*

To be rated as ‘6’ an index diagnosis should have both the following characteristics: (i) it should be completely different from the final diagnosis; and (ii) there should be compelling evidence documented to this effect.

- Example 1. A patient presented with ‘cramp like’ abdominal pain at the index consultation and a diagnosis of ‘UTI’ was made. Further review of the medical record revealed that several urine cultures were reported as negative, her symptoms persisted intermittently for many months, were relieved to a degree by Buscopan and subsequent ultrasound imagining found multiple gallstones. In this example, the index and final diagnosis are completely different, e.g. they relate to completely different anatomical systems (genito-urinary vs. digestive) *and* pathological processes (infection vs. inflammation). There is also ample evidence from multiple sources to confirm the accuracy of the final diagnosis.

Even after carefully reviewing a record, there may still not be sufficient evidence to evaluate the accuracy of some diagnoses. Consider the following two examples.

Example 1. The index diagnosis is ‘stress’. At first, it may seem very challenging to provide evidence that this diagnosis is accurate, given the diagnosis is also a symptom and the subjective nature of this symptom. However, if several clinicians entered this diagnosis, there is evidence that the patient attended over a period of time for this problem and there is documentation of eventual improvement, for example by the patient returning to work, this should all be considered as ‘evidence’.

Example 2. An index diagnosis of ‘viral illness’. The diagnosis may be accurate, but there may be little or no evidence for this if there are no other clinical encounters and if the index consultation’s clinical entry was scant on detail.

**Degree of confidence that a missed diagnostic opportunity occurred**

Reviewers should indicate their own confidence in their rating of the accuracy of each index diagnosis. The 6-point rating scale is: 1=Very confident; 2=confident; 3=somewhat confident; 4=a little uncertain; 5=uncertain; and 6=very uncertain.

The rating should reflect the confidence at the *end of the review*. In other words, if more than one reviewer is involved for *any* reason, the number to enter on the DEF should be the one that best reflects their final, collective confidence in the rating of the diagnostic accuracy. Consider the following example.

- Example 1. Reviewer DA finds some evidence that index diagnosis DXN1 may have been a missed diagnostic opportunity and rates the accuracy as a ‘5’. However, his confidence in his rating is very low (a ‘6’) and he decides to discuss the case with reviewer DE. Reviewer DE agrees that there was a missed diagnostic opportunity and that the accuracy should be ‘5’. After a lengthy discussion they agree that they are somewhat confident in this rating – a ‘3’. They decide to discuss the case with a member of the steering group. After further discussion the three reviewers confidently agree that a MDO with a rating of ‘5’ occurred. They indicate their confidence with a ‘1’.

**Impact of missed diagnostic opportunities on patients**

Reviewers should indicate the impact of each MDO on patients using a previously validated 4-point rating scale. Reviewers also have the option to select ‘unclear’ if there is not sufficient evidence in the record to make a judgment. The scale is described in more detail below and illustrated with examples.

*No harm (1)*

This rating includes ‘near misses’ and instances where harm was mitigated. The severity ratings of MDOs are dependent on individual patient characteristics and contexts.

- Example 1. An index diagnosis of a ‘incisional hernia’ was made in a patient who had recently undergone surgical resection of colon cancer. The patient subsequently had a specialist review and CT scan that excluded this diagnosis. In this example, the specialist review and CT scan had previously been arranged to evaluate the treatment of his cancer. The diagnostic error therefore did not cause the patient any inconvenience, e.g. additional or extra investigations or harm and its impact was coded as ‘1’. However, if an otherwise healthy patient had been referred with an index diagnosis of ‘hernia’ and was subsequently found not to have this problem, the impact should have been rated as ‘2’.

*Mild harm (2)*

This rating includes any MDO with an impact on patients that are judged to be minor or inconveniences, such as unnecessary follow-up appointments (in any setting), treatments and/or investigations. The impact of MDOs with a rating of ‘2’ should be transient and without any residual effect.

- Example 1. The index diagnosis was ‘iron deficiency’ and the patient was commenced on supplementary iron tablets. However, subsequent haematinics and FBC were normal. The patient was informed of the results and advised to stop the treatment. The reviewer coded the diagnostic error’s impact as ‘2’. However, if the patient had developed severe constipation resulting in an anal fissure as a result of the iron tablets, the impact of the diagnostic error should have been rated as ‘3’.

*Moderate harm (3)*

A pragmatic test of the severity of harm is for reviewers to consider how they would have felt if the impact had been on them personally. Moderate harm includes physical or psychological distress, but should always be self-limiting in nature and completely resolve without complication over time.

- Example 1. The index diagnosis was ‘soft tissue injury of foot’ while the final diagnosis several months later (confirmed through biochemistry) was ‘gout’. Further review of the record found that this patient had presented on several occasions with recurrent episodes of significant pain resulting in time off work. The reviewer therefore rated the impact as ‘3’. However, if the patient had developed gouty tophi after years of untreated gout, then the impact should have been rated as ‘4’.

*Severe harm (4)*

Any harm with prolonged or permanent impact on the patient. This category includes any preventable admission.

- Example 1. The index diagnosis was irritable bowel syndrome but the final diagnosis, many months later, was histological confirmed metastatic bowel cancer.

**Contributing factors to missed diagnostic opportunities**

Reviewers should try to identify the main contributing factors to any missed diagnostic opportunities. There are five main groups of factors, relating to: (1) the patient-provider encounter (history, exam, ordering tests/referrals based on assessment); (2) performance and interpretation of diagnostic tests; (3) follow-up and tracking of diagnostic information over time; (4) subspecialty and referral-specific issues; and 5) patient-related factors. Reviewers also have the option of ‘unclear’ (6) if there is not sufficient evidence in the record to make a judgement. A few selected examples of contributing factors are listed below.

*Patient-provider factors*

- The clinical entry recording the index consultation does not have sufficient detail or seem to omit clinical information that would normally have been expected. For example, an elderly patient’s index diagnosis is ‘hypertension’ but there is no record of a blood pressure.
- A potential knowledge and/or skill deficiency. For example, a patient presented with back pain during the index consultation. The clinician did not perform any physical examination. When his colleague examined the patient the next day she found evidence of shingles.
- A patient does not disclose a full or honest history. For example, a patient with presents with mild jaundice but denied alcohol abuse.

*Performance and interpretation of diagnostic tests*

- Missed Diagnostic Opportunities may result from problems related to the performance and interpretation of diagnostic tests. This commonly occurs when tests are ordered unnecessarily, when the wrong test is ordered, or when the test is inappropriate. Diagnostic tests may be misinterpreted by the lab or the radiologist or sometimes if the ordering diagnostician does not convey background information needed to interpret the test correctly.

*Follow-up and tracking of diagnostic information over time*

- Inadequate follow-up of diagnostic test results may occur if test result management systems do not communicate abnormal results to ordering clinicians in a timely manner; this is particularly problematic when the ordering clinician is not readily identified. Clinicians may also fail to communicate abnormal test results to their patients. Patients, in turn, may misunderstand the clinician’s follow-up instructions. For instance, a patient may not appreciate the importance of completing a “one-week rapid follow-up” visit scheduled by the clinician. Logistical barriers to effective follow up include protracted wait times for follow-up appointments, and ambiguity regarding the clinician (e.g. primary care provider vs. specialist) who is responsible for follow-up when more than one clinician is involved in the patient’s care.
- Example: Investigation results are not added to a patient’s clinical record. As a result, the clinician did not have access to relevant information during the index consultation.

*Subspecialty and referral-specific issues;*

- The subspecialty consultation process is another area vulnerable to diagnostic errors. Primary care physicians may fail to order a consultation despite clear reasons to do so. Consultants, in turn, may fail to communicate their recommendations to the primary care physician. There is sometimes ambiguity regarding the clinician who is responsible for implementing the consultant’s recommendation. Indeed, information must flow freely between the referring and consulting physicians to ensure that both are well informed, especially when patients refer themselves to specialists. Clinicians often gain useful information from consultants about difficult-to-diagnose cases, but may be disappointed when the consultant does not meet their expectations. Consultants may fail to address the issue faced by the referring clinician, or may be confused about their role in the case. Conflicting diagnoses from different consulting physicians may be difficult for the primary care provider to adjudicate.
- Example. Delayed discharge documentation meant the clinician did not have access to relevant information during the index consultation.

*Patient-related factors*

- Patient-related behaviours—such as care-seeking and adherence. Cognitive impairments, language issues and health literacy are a significant and underestimated problem, in which some patients are not able to communicate effectively with their clinicians. They may not be able to relay symptoms accurately, or to understand physicians’ recommendations. Problems may also result from patient non-adherence to pre-test instructions (e.g., fasting status or bowel preparation) or failure to show up for the scheduled test.

*Confounding factors*

- Many long-term conditions cause symptoms and signs which can be difficult to distinguish from new problems. For example, a patient known to have asthma and COPD presented with worsening cough and dyspnoea. The index diagnosis was ‘uncontrolled asthma’ but an ECHO several months later found ‘heart failure’.

If the ‘confounding factors’ option is selected, the specific confounding factors should briefly be described in the box provided for this purpose.

**Description of missed diagnostic opportunities**

Reviewers should summarize each MDO in this section. There should be sufficient information to allow a member of the steering group to understand what happened and the quality of the evidence for a MDO.

**Reviewer comments**

Reviewers are encouraged to document practical challenges, personal reflections, reminders or any other information that they consider may potentially be useful for analysing the data in this section.

Finally, in cases where the two reviewers differ, a separate DEF form will be completed to record the agreed decision (either after discussion or when referred to the steering group). This form will also record in the reviewers notes what the main reasons were for a difference in the first place, what the basis for reaching an agreement was (e.g. did one reviewer miss something in the notes); and which reviewer’s decision was taken as the agreed one.

1. 1. Box 1 was adapted with permission from: Bowie P, de Wet C. Safety and Improvement in Primary Care: the essential guide. Radcliffe Publishing Ltd. 2014: London.

   [↑](#footnote-ref-1)
