# Supplementary material for: Missed diagnostic opportunities and English general practice: a study to determine their incidence, confounding and contributing factors and potential impact on patients through retrospective review of electronic medical records
Source: Implement Sci. 2015 Jul 29;10:105. doi: 10.1186/s13012-015-0296-z (PMC4518650; doi:10.1186/s13012-015-0296-z)
Supplement: Additional file 1: — Project introductory letter. (DOCX 70 kb) [file 13012_2015_296_MOESM1_ESM.docx]

|  | **NIHR Greater Manchester Primary Care**  **Patient Safety Translational Research Centre** |
| --- | --- |
|  | The University of Manchester  Williamson Building, Oxford Road  Manchester, M13 9PL  Tel: 0161 275 7642  Web: [www.gmpstrc.nihr.ac.uk](http://www.gmpstrc.nihr.ac.uk) |

Dear Dr [name],

**Why are we contacting you?**

We are contacting you to inform you of a study we are conducting and would like for your practice to participate in. We are studying the topic of ‘Missed Diagnostic Opportunities’ in English general practice. The concept of a missed diagnostic opportunity essentially implies that something different could have been done to make the correct diagnosis earlier or that the diagnosis was incorrect. We are interested in looking at patient records to explore the processes that contribute to such missed diagnostic opportunities as well as any resulting impact(s). We hope that the study will provide useful insights into diagnosis in general practice which can then be used in the development of training and educational materials.

The study has received a favourable ethical review from an NHS Research Ethics Committee – Ref: and had also been endorsed by the clinical lead of your CCG [insert name].

**What do I/we need to do?**

If your practice agrees to participate you will not be asked to do anything directly, but simply to provide access and space for our practice level study team (an administrator plus two GP reviewers) to conduct the study. Specifically, we will provide a trained administrator to locate the necessary 100 patient records (which meet the inclusion criteria) from your system. Once this list of 100 patient records has been generated and stored on-site by the administrator, two GP reviewers will visit the practice on separate occasions and each will independently work through the list generated by our administrator. The reviewers will be using a structured data collection tool and will not be collecting any patient identifiable information other than age and gender. We anticipate that the study will require approximately 5 days of access for the practice level study team.

**What can we offer you for participating?**

We are able to pay each practice £1000. Furthermore, we can feedback on the findings that relate to your individual practice.

**Interested in finding out more?**

Please read the enclosed information sheet. If you have any further questions please contact one of the people listed on enclosed the information sheet. We will be sending out two further contacts to allow ample opportunity to participate. Please indicate on the attached expression of interest form if you would prefer not to receive any further communications regarding this study.

Sincerely,

On behalf of the Research Team:

Dr Sudeh Cheraghi-Sohi	Professor Stephen Campbell	Professor Aneez Esmail
